# Hormonal Contraception and Endometrial Thickness in IVF/ICSI Cycles: A Multicentre Historical Cohort Study

**DOI:** 10.1111/1471-0528.18295

**Published:** 2025-07-16

**Authors:** Mette Peters Michaelsen, Laura Cæcilie Nielsen, Michelle Poulsen, Regitze Gyldenholm Skals, Bettina Troest, Janne Gasseholm Bentzen, Jimmi Elers, Anette Vestergaard Gabrielsen, Marie Louise Grøndahl, Betina Boel Povlsen, Mikael Tang‐Pedersen, Ulrik Schiøler Kesmodel

**Affiliations:** ^1^ Department of Obstetrics and Gynaecology Aalborg University Hospital Aalborg Denmark; ^2^ Department of Health Science and Technology Aalborg University Aalborg Denmark; ^3^ Research Data and Biostatistics Aalborg University Hospital Aalborg Denmark; ^4^ The Fertility Unit, Department of Obstetrics and Gynaecology Aalborg University Hospital Aalborg Denmark; ^5^ The Fertility Clinic, Department of Gynaecology, Fertility, and Obstetrics, Rigshospitalet Copenhagen University Hospital Copenhagen Denmark; ^6^ TFP Stork Fertility A/S Copenhagen Denmark; ^7^ The Fertility Clinic Horsens Regional Hospital Horsens Denmark; ^8^ The Fertility Clinic Herlev and Gentofte Hospital Herlev Denmark; ^9^ The Fertility Clinic Skive Regional Hospital Skive Denmark; ^10^ The Fertility Clinic Odense University Hospital Odense Denmark; ^11^ Department of Clinical Medicine Aalborg University Aalborg Denmark

**Keywords:** art, contraception, endometrial thickness, endometrium, levonorgestrel intrauterine system

## Abstract

**Objective:**

To study the association between previous use of levonorgestrel intrauterine system (LNG‐IUS) and endometrial thickness (EMT) in women undergoing in vitro fertilisation (IVF)/intracytoplasmic sperm injection (ICSI) cycles.

**Design:**

Multicentre historical cohort study.

**Setting:**

Eight Danish public and private fertility clinics.

**Population:**

12786 women aged 18–46 years contributing with an EMT measurement from 22 464 different IVF/ICSI treatment cycles between 2000 and 2021.

**Methods:**

Exposure was previous use of LNG‐IUS, combined oral contraceptive pills (OCPs), progeste‐only pills (POPs), no/other contraception or combined, cumulated use of contraception when more contraceptives had been used during the inclusion period. Further, ever use of LNG‐IUS was categorised into 0–3 years, > 3–6 years, > 6–9 years and > 9 years. Mixed effect logistic regression adjusted for age, BMI, smoking, educational level, total FSH dose and fertility clinic was used.

**Main Outcome Measure (s):**

EMT (< 7 mm ≥ 7 mm).

**Results:**

Statistically significantly higher odds of EMT ≥ 7 mm were found for OCPs [odds ratio (OR) 3.53 (95% confidence interval (95% CI) 1.29–9.65)], POPs [OR 6.43, (95% CI 1.45–28.63)] and no/other contraception [OR 6.67, (95% CI 2.37–18.74]) relative to LNG‐IUS in IVF/ICSI cycles. Further, all duration categories of ever use of LNG‐IUS were associated with statistically significantly lower odds of obtaining an EMT ≥ 7 mm compared to no/other contraception.

**Conclusions:**

In this study, previous use of LNG‐IUS was associated with decreased endometrial growth in women undergoing IVF/ICSI.

## 1 Introduction

Long‐acting reversible contraceptive methods are among the most effective contraceptives available today and are an advantageous choice of contraception for women who wish to conceive in the future [1, 2]. Among these is the levonorgestrel intrauterine system (LNG‐IUS), the use of which has increased in recent years [3, 4, 5]. The LNG‐IUS releases levonorgestrel locally, causing the cervical mucus to thicken, thereby decreasing spermatozoa motility, which creates a hostile environment for the spermatozoa. Further, it causes atrophy of the endometrium. The combination of these effects prevents fertilisation and implantation [3, 6]. As LNG‐IUS is a reversible contraceptive method, removal should result in a return to baseline fertility. However, current literature on this topic is contradicting. While some studies have shown that the use of LNG‐IUS does not affect the return to baseline/normal fertility after removal [7], others have found delayed conception after removal [3, 8]. Endometrial development plays an essential role in achieving pregnancy, as a sufficient endometrial thickness (EMT) is needed for successful embryonic implantation into the uterine wall [9]. A previous study investigated the effect of combined oral contraceptive pill (OCP) use on the endometrium and found that long‐term use of OCPs may have a negative effect on endometrial growth [9]. Although the sample size in the study was small, the findings suggest a possible unidentified adverse side effect of using OCPs. As OCPs and LNG‐IUSs are both hormonal contraceptive methods containing progestin, it can be hypothesised that the use of LNG‐IUS may also affect endometrial growth and thereby fertility. Consequently, the objective of this study was to investigate whether previous use of the LNG‐IUS negatively influences the EMT in women undergoing in vitro fertilisation (IVF)/intracytoplasmic sperm injection (ICSI) treatment.

## 2 Methods

This study was a register‐based multicentre historical cohort study. The study has been reported in accordance with Strengthening the Reporting of Observational Studies in Epidemiology (STROBE) [10].

### 2.1 Study Population and Data Sources

Data from women aged 18–46, who had undergone at least one IVF/ICSI treatment cycle and hence had at least one measurement of EMT, were obtained from 1 January 2000 to 31 December 2021. Information on previous contraceptive use was obtained from 1995 onwards. Women with conditions known to affect endometrial thickness, that is, Asherman's syndrome, transcervical resection of the endometrium and septum resection were excluded (The Danish Health Care Classification System (SKS) codes: KLCB28, KLCA16, KLCB25, KLCB32, KLCC05, KLCB22, KLCB98, KLCW98, KLCG02, KLCG98 and DN856). Women with a BMI of < 15 and ≥ 40, a total follicle stimulating hormone (FSH) dose of > 8000 IU, and an EMT of < 1.5 mm or > 20 mm were also excluded to avoid misclassification. Further, women undergoing treatment cycles other than IVF/ICSI were excluded, as were women with only a temporary Danish Personal Identification Number (CPR). Historical data from eight Danish public and private fertility clinics using the fertility database Danish Medical Data Center (DMDC) were collected electronically and combined with data from the Danish National Patient Register (DNPR) [11], the Danish National Prescription Register (NPR) [12] and the Danish Education Register (DER) [13] using each individual's unique CPR to link data.

### 2.2 Clinical Setting

All eight fertility clinics are certified European Union Tissue Directive Centres (Table S1). All laboratories at the participating fertility clinics follow state‐of‐the‐art techniques for IVF, ICSI and associated techniques such as culturing, evaluation of gametes and embryos and cryopreservation. For IVF and ICSI treatments, either long agonist or short antagonist protocol was used with FSH or menotropin. When the leading 2–3 follicles reached 17–18 mm, final maturation was induced with hCG or agonist trigger, and oocyte retrieval was performed after 34–36 h. Progesterone supplements were given as luteal phase support, and embryo transfer was performed after 2, 3 or 5 days, depending on calendar time period.

#### 2.2.1 Exposure

Method of contraception was obtained from DNPR and NPR and was categorised into five variables: LNG‐IUS (ATC code: G02BA03; SKS codes: MG02BA03, BJCD01, BJCZ01, BJCZ1 and BJCZ2); OCPs (ATC codes: G03AA07, G03AA079, G03AA10, G03AA11, G03AA12, G03AA14, G03AA16, G03AA18, G03AB03, G03AB05 and G03AB08; SKS code: BJCA0); progesti‐only pills (POPs) (ATC codes: G03AC01, G03AC09 and G03AC10; SKS code: BJCA1); no/other contraception and combined, cumulated use of contraception (CC). The CC variable was used when multiple contraceptives had been used either simultaneously or sequentially during the inclusion period, while the remaining categories were used when only a single type of contraception had been used throughout the study period. A sixth categorisation with copper intrauterine device (IUD) (SKS codes: MG02BA02, BJCD00, BJCZ00, BJCZ1 and BJCZ2) was planned; however, due to too few observations, these were excluded. Further, ever use of LNG‐IUS defined as either the use of LNG‐IUS only or as a part of a cumulative exposure to contraceptives during the inclusion period was categorised as 0–3, > 3–6, > 6–9 and > 9 years of previous ever use.

#### 2.2.2 Outcome

The primary outcome EMT was obtained from DMDC and categorised into < 7 and ≥ 7 mm. The last EMT measurement before oocyte retrieval was used as the outcome, or—in the case of a started but subsequently cancelled cycle—the last measurement before cancellation. The secondary outcome was clinical pregnancy defined as a confirmed intrauterine pregnancy at gestational week 7–8 visualised by transvaginal ultrasound.

#### 2.2.3 Covariates

A Directed Acyclic Graph was used to identify relevant potential confounders (Figure S1). These included age (18–22, > 22–26, > 26–30, > 30–34, > 34–38, > 38–42, > 42–46 years, categorical/continuous), body mass index (BMI; 15–18.4, ≥ 18.5–24.9, ≥ 25.0–29.9, ≥ 30.0–34.9, ≥ 35.0–39.9 kg/m^2^ categorical/continuous), smoking (yes/no, categorical), total dose of FSH (units; IU, continuous) obtained from DMDC and educational level (low/middle/high according to International Standard Classification of Education 2011 (ISCED‐11), categorical) obtained from DER. Further, fertility clinic (1–8, categorical) obtained from DMDC and length of previous contraceptive use (0–3 years, > 3–6 years, > 6–9 years, > 9 years, categorical) obtained from DNPR and NPR were included as covariates. Information on covariates was collected at each cycle. When information on both prescription, insertion and removal of an IUD was available, the exact length of use was applied. If information on removal was not available but a new insertion took place 5 years later, it was assumed that the women had used the IUD for the maximum recommended time for that IUD. When only information on prescription or insertion was available, an estimated length of use of 4 years was used based on scientific literature [14, 15, 16] and knowledge on general use of IUDs in Denmark [17]. When only an unspecific insertion or removal code was available and no ATC code was available, the IUD was assumed to be a copper IUD. Length of use of oral contraceptives was identified based on prescriptions. A cessation of oral contraceptives was defined as a minimum of 4 weeks. All information on contraceptive use from 1995 until the initiation of first medically assisted reproductive (MAR) treatment was accumulated as time on contraception.

### 2.3 Statistical Analysis

#### 2.3.1 Descriptives

Histograms were used to test for normality. For normally distributed data, means and standard deviations (SD) were calculated. For non‐normally distributed data, medians and 10th and 90th percentiles were calculated. Categorical data are presented as frequencies and percentages.

#### 2.3.2 Data Analysis

All analyses were conducted using mixed effects logistic regression models. Multiple outcome measurements were included for each woman with a measurement of EMT corresponding to one cycle of MAR treatment, and therefore, the ID of the women were included as a random effect in all regression models. An analysis was conducted to analyse the association between previous methods of contraception and EMT. LNG‐IUS was used as the reference group, thereby allowing for a comparison to other contraceptive methods. This analysis was conducted both with and without the exposure group no/other contraception in order to investigate the influence of adjusting for time of previous contraceptive use. The association between ever use of LNG‐IUS and EMT was tested by categorising LNG‐IUS into 0–3, > 3–6, > 6–9 and > 9 years of ever use. No/other previous use of contraception was used as the reference group. A sensitivity analysis was performed to evaluate whether a potential difference in EMT between contraceptive methods was more pronounced when EMT values close to the chosen 7 mm cut‐off value for thin endometrial lining were excluded. In this analysis, EMT was dichotomised into ≤ 5 or ≥ 7 mm. LNG‐IUS at 0–3, > 3–6, > 6–9 and > 9 years of ever use was used as exposure groups, and no/other contraception was used as the reference group. Secondary analyses were performed using clinical pregnancy as the outcome. Here, only completed cycles where embryo transfer had been performed were included. Secondary analyses were conducted using both the different contraceptive methods as exposure with LNG‐IUS as the reference and the different duration categories of ever use of LNG‐IUS with no/other contraception as the reference, respectively. Post hoc analyses restricted to the first treatment cycle of each individual were conducted to assess whether the use of multiple‐cycle data could have influenced the results. These post hoc analyses mirrored the analyses assessing the association between previous methods of contraception and EMT and ever use of LNG‐IUS and EMT, respectively. Assumptions of a linear relationship between continuous independent variables and logit of the dependent variable and no multi‐collinearity between independent variables were assessed using the Box Tidwell test and variance inflation factor, respectively. All results are presented as both crude and adjusted odds ratios (OR) with 95% confidence intervals (CIs). ORs were reported due to the use of mixed effect logistic regression models. All analyses were adjusted for age, BMI, smoking status, educational level, total dose of FSH, fertility clinic and when appropriate time of previous contraceptive use. Covariates were used either continuously or categorically depending on whether they fulfilled the relevant assumptions. All analyses were carried out in StataCorp. 2023. *Stata Statistical Software: Release 18*. College Station, TX: StataCorp LLC.

## 3 Results

**FIGURE 1:**
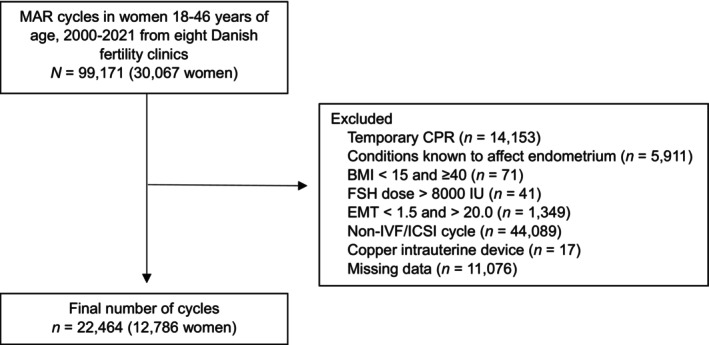
Flow chart of the cohort selection process. BMI, body mass index; CPR, personal identification number; EMT, endometrial thickness; FSH, follicle stimulating hormone; IVF/ICSI, in vitro fertilisation/intracytoplasmic sperm injection; MAR, medically assisted reproduction.

From the 99 171 treatment cycles initially identified, a final study population of 12 786 individuals contributing a total of 22 464 treatment cycles was eligible for analysis, each contributing the last EMT measurement in one treatment cycle (Figure 1). Age, BMI, smoking status and educational level were comparable with respect to included and excluded cycles (Table S2). Sample sizes within each exposure group varied, with LNG‐IUS being the smallest with 76 cycles and OCPs being the largest with 14 535 cycles. In the included cycles, the median age was 32.67 years, and the median BMI was 23.77 kg/m^2^. In all exposure groups, non‐smoking was more frequent than smoking, with > 90% of all cycles being non‐smokers, and 54.1% had a high level of education. The median total dose of FSH was 2000 IU. EMT < 7 mm varied, with 4.8% of cycles in the no/other contraception group as the lowest and 14.5% in the LNG‐IUS group as the highest (Table 1).

**TABLE 1:** Characteristics corresponding to cycles included.

	LNG‐IUS	CC	OCPs	POPs	No/Others	Total
*N* (%)	76 (0.3)	4899 (21.8)	14 535 (64.7)	136 (0.6)	2818 (12.5)	22 464 (100.0)
Age, median (10th–90th percentiles)	32.22 (26.22; 38.84)	32.50 (26.58; 39.47)	32.42 (26.69; 39.53)	32.65 (25.91; 40.14)	34.36 (27.59; 39.85)	32.67 (26.73; 39.56)
BMI, median (10th–90th percentiles)	23.66 (20.51; 31.17)	23.88 (19.94; 30.10)	23.78 (19.72; 30.10)	24.06 (19.04; 29.37)	23.50 (19.57; 30.09)	23.77 (19.78; 30.10)
Smoking, *n* (%)						
No	(> 90)^a^	4443 (90.7)	13 275 (91.3)	123 (90.4)	2582 (91.6)	(> 90)^a^
Yes	(< 10)^a^	456 (9.3)	1260 (8.7)	13 (9.6)	236 (8.4)	(< 10)^a^
Educational level, *n* (%)						
Low	13 (17.1)	369 (7.5)	1031 (7.1)	18 (13.2)	499 (17.7)	1930 (8.6)
Medium	27 (35.5)	1913 (39.0)	5517 (38.0)	42 (30.9)	888 (31.5)	8387 (37.3)
High	36 (47.4)	2617 (53.4)	7987 (55.0)	76 (55.9)	1431 (50.8)	12 147 (54.1)
FSH, median (10th–90th percentiles)	1900.00 (140.40; 3611.25)	2000.00 (925.00; 3600.00)	1987.00 (925.00; 3600.00)	1835.00 (510.00; 4072.50)	2025.00 (900.00; 3600.00)	2000.00 (913.00; 3600.00)
EMT, *n* (%)						
< 7 mm	11 (14.5)	456 (9.3)	1058 (7.3)	7 (5.1)	135 (4.8)	1667 (7.4)
≥ 7 mm	65 (85.5)	4443 (90.7)	13 477 (92.7)	129 (94.9)	2683 (95.2)	20 797 (92.6)
Number of women (%)	50 (0.4)	2932 (22.9)	8116 (63.5)	83 (0.6)	1605 (12.6)	12 786 (100.0)

Abbreviations: CC, combined, cumulated use of contraception; EMT, endometrial thickness; FSH, follicle‐stimulating hormone; LNG‐IUS, levonorgestrel intrauterine‐system; OCPs, combined oral contraceptive pills; POPs, progestin‐only pills.

^a^ Exact number of cycles not shown to protect confidentiality.

### 3.1 Association Between Method of Contraception and Endometrial Thickness

Analysis on the association between previous method of contraception and EMT adjusted for age, BMI, smoking, educational level, total dose of FSH and fertility clinic showed statistically significantly increased odds of an EMT ≥ 7 mm in cycles with previous OCP use, previous POP use and no/other previous use of contraception relative to LNG‐IUS. No statistically significant difference in odds was seen for previous CC use (Table 2).

**TABLE 2:** Analysis assessing the association between previous method of contraception and odds of achieving endometrial thickness ≥ 7 mm.

	*N*	OR	95% CI	OR^a^	95% CI^a^
LNG‐IUS	76	1.00	Reference	1.00	Reference
CC	4899	2.19	(0.80; 6.05)	2.38	(0.87; 6.54)
OCPs	14 535	3.15	(1.15; 8.63)	3.53	(1.29; 9.65)
POPs	136	5.77	(1.29; 25.77)	6.43	(1.45; 28.63)
No/other	2818	5.63	(2.00; 15.87)	6.67	(2.37; 18.74)

*Note:* Mixed effect logistic regression was used, and personal identification number (CPR) of each woman was included as a random effect.

Abbreviations: 95% CI, 95% confidence interval; CC, combined, cumulated use of contraception; LNG‐IUS, levonorgestrel intrauterine‐system; OCP, combined oral contraceptive pill; OR, odds ratio; POP, progestin‐only pill.

^a^ Adjusted for age (continuous), BMI (continuous), smoking, educational level, total dose of follicle stimulating hormone (continuous) and fertility clinic.

When also adjusting for time of previous contraceptive use, previous CC use, previous OCP use and previous POP use all showed statistically significantly higher odds of an EMT ≥ 7 mm relative to LNG‐IUS (Table S3).

### 3.2 Association Between Ever Use of LNG‐IUS and Endometrial Thickness

The adjusted analysis assessing the association between ever use of LNG‐IUS and EMT showed that 0–3 years, > 3–6 years and > 6–9 years and > 9 years of ever LNG‐IUS use were all associated with a statistically significantly lower odds of having an EMT ≥ 7 mm relative to no/other contraception (Table 3).

**TABLE 3:** Analysis assessing the association between ever use of LNG‐IUS and odds of achieving an endometrial thickness ≥ 7 mm.

	EMT ≥ 7 mm, *n* (N)	OR	95% CI	OR^a^	95% CI^a^
No/other	2683 (2818)	1.00	Reference	1.00	Reference
Ever use LNG‐IUS 0–3 years	1202 (1329)	0.35	(0.23; 0.52)	0.32	(0.21; 0.48)
Ever use LNG‐IUS > 3–6 years	884 (1036)	0.19	(0.13; 0.29)	0.18	(0.12; 0.27)
Ever use LNG‐IUS > 6–9 years	65 (78)	0.13	(0.05; 0.35)	0.11	(0.04; 0.31)
Ever use LNG‐IUS > 9 years	24 (31)	0.07	(0.02; 0.30)	0.05	(0.01; 0.21)

*Note:* Mixed effect logistic regression was used, and personal identification number (CPR) of each woman was included as a random effect.

Abbreviations: 95% CI, 95% confidence interval; LNG‐IUS, levonorgestrel intrauterine‐system; OR, odds ratio.

^a^ Adjusted for age (continuous), BMI (categorical), smoking, educational level, total dose of follicle stimulating hormone (continuous) and fertility clinic.

### 3.3 Sensitivity Analysis

Results of the sensitivity analysis showed that all durations of ever use of LNG‐IUS remained statistically significantly associated with lower odds of having an EMT ≥ 7 mm after changing the cut‐off value for EMT from < 7 mm and ≥ 7 mm to ≤ 5 mm and ≥ 7 mm (Table S4).

### 3.4 Secondary Analyses

Adjusted secondary analyses on the association between previous methods of contraception and clinical pregnancy showed no statistically significant difference in the odds of achieving a clinical pregnancy in cycles when comparing LNG‐IUS to other contraceptive methods with or without adjusting for time of previous contraceptive use (Table S5). Additionally, adjusted secondary analyses showed no statistically significant difference in the odds of achieving a clinical pregnancy in any of the duration categories of ever use of LNG‐IUS relative to no/other contraception (Table S6).

### 3.5 Post Hoc Analyses

Adjusted post hoc analyses restricted to the first treatment cycle assessing the association between previous method of contraception and EMT showed non‐significant increased odds of an EMT ≥ 7 mm for all contraceptive methods relative to LNG‐IUS both with and without adjusting for time of previous contraceptive use (Table S7). Adjusted post hoc analyses assessing the association between ever use of LNG‐IUS and EMT showed statistically significantly lower odds of an EMT ≥ 7 mm for all duration categories relative to no/other contraception (Table S8).

## 4 Discussion

### 4.1 Main Findings

In this study, previous use of LNG‐IUS was associated with statistically significantly lower odds of obtaining an EMT ≥ 7 mm when compared to other contraceptive methods in IVF/ICSI cycles. Further, we found a statistically significant association between ever use of LNG‐IUS and a thin endometrium compared to no/other previous use of contraceptives regardless of the duration of use.

### 4.2 Strengths and Limitations

Our study has several methodological strengths. National registers and medical records were used as the data source, allowing rather large sample sizes. Further, all variables included in this study represent relatively objective measures. Additionally, EMT measurements were measured in the same way throughout the study period using transvaginal ultrasound, which strengthens the precision of the outcome. Although the exposure occurred prior to fertility treatment, the current study assessed a clinically relevant research question in a clearly defined population. Restricting eligibility to the start of contraceptive use would be neither clinically meaningful nor feasible. Therefore, the risk of selection bias due to the time of the exposure is considered to be minimal. It should also be acknowledged that this study has limitations, which should be considered when interpreting the results. Since data obtained from registers and medical records is reported by different professionals, departments and at different time points, this might result in some variation in coding. Potential unmeasured confounding could be present as we were unable to control for all possible confounders such as genetics and reproductive history, which could have biased the results. Additionally, missing data was handled by complete case analysis, which may have introduced some selection bias. Another limitation is the small sample size within the exposure group of those who had only used LNG‐IUS and the disproportionality compared to other contraceptive methods. Further, when divided into different duration categories of ever use of LNG‐IUS, previous durations of 6–9 years and > 9 years also had notably smaller sample sizes. This may have negatively impacted the statistical power of the results and should be considered in the interpretation of the findings as the results may likely be hypothesis‐generating rather than definitive.

### 4.3 Interpretation

While most other studies investigating female fertility after contraceptive use have focused on a possible delay in fertility after the use of contraceptive methods [3, 7, 8], only a few have investigated EMT. The effect of OCPs use on EMT has previously been investigated by Talukdar et al. [9]. In their study, patients undergoing FET cycles more often had an EMT < 7 mm if they had a previous history of OCP use of 5 years or more. In another study by Homminga et al. [18], it was investigated whether a thin EMT was more frequent in patients undergoing preimplantation genetic testing for monogenic disorders during fertility treatment, and whether this was related to previous contraceptive use. They reported a non‐significant trend towards a higher probability of having an EMT < 8 mm in women with a history of LNG‐IUS use compared to OCPs, Cu‐IUDs, depot medroxyprogesterone acetate and vaginal ring. These results are in agreement with this current study's findings of former LNG‐IUS users having statistically significantly higher odds of having a thinner endometrium compared to other contraceptive methods in IVF/ICSI cycles. The analyses assessing the association between previous method of contraception and EMT showed wide 95% CIs, which could indicate that the sample sizes in one or more exposure groups were too small to conduct a reliable analysis.

When assessing the association between different durations of ever use of LNG‐IUS and EMT, we found that those who had no/other previous use of contraceptives had statistically significantly higher odds of having an EMT ≥ 7 mm compared to those who had ever used an LNG‐IUS. In this analysis, the narrow 95% CIs indicate a higher precision, which might be due to more observations in each exposure group, as all previous users of LNG‐IUS regardless of use of other contraceptives during the period were included. The exposure group no/other included those who had never used LNG‐IUSs, OCPs and POPs. However, it is not known whether this group had previously only used barrier methods or other contraceptives containing hormones, for example, vaginal ring. Our sensitivity analysis showed that the odds of obtaining an EMT of ≥ 7 mm remained statistically significantly decreased when changing the cut‐off value for EMT. These results suggest that a negative effect of LNG‐IUS on EMT is present.

A potential source of bias is the use of multiple‐cycle data [19]. To address this, post hoc analyses restricted to the first treatment cycle were conducted. All post hoc analyses were directionally consistent with the primary analyses although the analyses comparing previous use of LNG‐IUS to other contraceptive methods were non‐significant, possibly due to the reduced sample size. These analyses suggest that the inclusion of multiple cycles did not substantially influence the results.

In this study, EMT was evaluated after the use of LNG‐IUS in women undergoing IVF/ICSI as a potentially relevant predictor of success. Although controversies remain on a specific value of EMT for successful implantation, it is generally considered that an EMT of minimum of 7 mm assessed by ultrasonographic measurement is required [9]. However, the clinical relevance of this threshold in relation to pregnancy‐related outcomes remains uncertain [20, 21]. In the secondary analyses assessing clinical pregnancy as the outcome, no statistically significant differences were seen after neither LNG‐IUS compared to other contraceptive methods nor no/other contraception compared to different durations of ever use of LNG‐IUS, respectively. This could indicate that the potential negative effect of LNG‐IUS on EMT does not have a negative impact on pregnancy‐related outcomes. However, the secondary analyses were restricted to a subpopulation including only non‐cancelled cycles, which should be considered. Future studies should aim to evaluate the association between LNG‐IUS‐induced changes in EMT and clinical outcomes such as pregnancy, live birth and miscarriage.

The biological explanation for a possible, altered endometrial growth after the use of LNG‐IUS is yet to be established. A proposed rationale for this is a downregulation of oestrogen receptors caused by prolonged exposure to progestin. It could be speculated that prolonged use of LNG‐IUS results in a downregulation of oestrogen receptors, which would result in a deteriorated proliferation of the endometrium, thereby inhibiting the development of a sufficient EMT for implantation [3, 18]. Because of its atrophic effect on the endometrium, LNG‐IUS is also indicated as treatment for endometrial hyperplasia, dysfunctional bleeding and endometriosis. Further, a reduced risk of endometrial cancer has been linked to the use of contraceptives containing progestins due to its antiproliferative effects on the endometrium. In addition to a reduced EMT, endometrial atrophy is also characterised by the loss of endometrial glands [3, 9]. It is possible that these effects on the endometrium may persist after discontinuation of LNG‐IUS, thereby causing an impaired ability to grow a sufficient endometrium and loss of secretions from endometrial glands that may affect the implantation and maintenance of pregnancy. However, more research is needed to elucidate the biological basis of the prolonged effect of LNG‐IUS on EMT after termination of use.

The findings of this study imply the possibility of LNG‐IUS use having a negative effect on endometrial growth. The LNG‐IUS is a popular method of contraception due to its high effectiveness and low maintenance. Despite the high use of contraceptive methods, research has shown that some women show concerns about the possibility that the use of contraceptives might have a negative impact on their fertility [22, 23]. Although studies investigating the effect of contraceptives on return to fertility following termination of use have not found an association, the possibility of LNG‐IUS negatively affecting thEMT cannot be dismissed. More knowledge on whether LNG‐IUS has an adverse effect on the endometrium is warranted, so women can make an informed decision as to what type of contraception they prefer to use.

## 5 Conclusion

In conclusion, this study showed that previous use of LNG‐IUS might have a negative impact on the EMT in IVF/ICSI cycles. However, we acknowledge that this present study has limitations, which should be considered when interpreting the results. Future research should accommodate these limitations to create evidence‐based knowledge on the potential prolonged negative impact of LNG‐IUS on the endometrium and its receptivity in IVF/ICSI cycles.

## Author Contributions

M.P.M., L.C.N. and U.S.K. contributed to study conceptualization and study design. M.P.M., L.C.N., M.P., B.T., J.G.B., J.E., A.V.G., M.L.G., B.B.P., M.T.P. and U.S.K. contributed to data curation. Data analysis was conducted by M.P.M., L.C.N. and R.G.S. M.P.M. wrote the original draft. All authors reviewed and edited the paper and approved the final version. Funding was obtained by M.P.M., L.C.N., M.P. and U.S.K.

## Ethics Statement

The study was registered with the North Denmark Region in agreement with the General Data Protection Regulation (22 November 2022, ID F2022‐136). In Denmark, register‐based research does not require approval from the ethics committees or patient consent. Approval to access medical records in the fertility database Danish Medical Data Center (DMDC) was obtained through the North Denmark Region (11 October 2022, Case no: 2022‐036879). Data were handled in Statistics Denmark (DST) in accordance with Danish legislation. In order to gain access to NPR through DST, approval was obtained from The Danish Health Data Authority (9 December 2022, FSEID‐00006398).

## Conflicts of Interest

B.B.P. is a board member of the Danish Fertility Society. M.L.G. has received consulting fees from Cooper Surgical and honoraria for teaching from Merck. U.S.K. has received funding from Gedeon Richter Nordic, Merck and IBSA for studies outside this work; honoraria for teaching from Tillotts Pharma AB and Merck; and travel support and conference expenses from Merck. The remaining authors have no conflicts of interest.

## Supporting information


**Table S1.** List of fertility clinics participating in the study.
**Table S2.** Characteristics of included and excluded cycles.
**Table S3.** Analysis assessing the association between previous method of contraception and odds of achieving an endometrial thickness ≥ 7 mm.
**Table S4.** Sensitivity analysis assessing whether a change in cut‐off values of endometrial thickness to ≤ 5 mm and ≥ 7 mm would affect the results.
**Table S5.** Secondary analyses assessing the association between previous method of contraception and odds of achieving a clinical pregnancy.
**Table S6.** Secondary analyses assessing the association between ever use of LNG‐IUS and odds of achieving a clinical pregnancy.
**Table S7.** Post hoc analyses assessing the association between previous method of contracepton and odds of achieving an endometrial thickness ≥ 7 mm using only cycle 1 data.
**Table S8.** Post hoc analyses assessing the association between ever use of LNG‐IUS and odds of achieving an endometrial thickness ≥ 7 mm using only cycle 1 data.
**Figure S1.** Directed acyclic graph showing potential confounding factors of the association between the use of contraceptives and endometrial thickness (EMT).

## Data Availability

In compliance with Danish legislation, data used in this study cannot be shared as it contains personal information.
