# Supplementary material for: Hormonal Contraception and Endometrial Thickness in IVF/ICSI Cycles: A Multicentre Historical Cohort Study
Source: BJOG. 2025 Jul 16;132(11):1681–8. doi: 10.1111/1471-0528.18295 (PMC12411652; doi:10.1111/1471-0528.18295)
Supplement: Supplementary file 1 — Table S1. List of fertility clinics participating in the study. Table S2. Characteristics of included and excluded cycles. Table S3. Analysis assessing the association between previous method of contraception and odds of achieving an endometrial thickness ≥ 7 mm. Table S4. Sensitivity analysis assessing whether a change in cut‐off values of endometrial thickness to ≤ 5 mm and ≥ 7 mm would affect the results. Table S5. Secondary analyses assessing the association between previous method of contraception and odds of achieving a clinical pregnancy. Table S6. Secondary analyses assessing the association between ever use of LNG‐IUS and odds of achieving a clinical pregnancy. Table S7. Post hoc analyses assessing the association between previous method of contracepton and odds of achieving an endometrial thickness ≥ 7 mm using only cycle 1 data. Table S8. Post hoc analyses assessing the association between ever use of LNG‐IUS and odds of achieving an endometrial thickness ≥ 7 mm using only cycle 1 data. Figure S1. Directed acyclic graph showing potential confounding factors of the association between the use of contraceptives and endometrial thickness (EMT). [file BJO-132-1681-s001.docx]

**Supplementary figures and tables**

***Hormonal contraception and endometrial thickness in IVF/ICSI cycles: a multicentre historical cohort study***


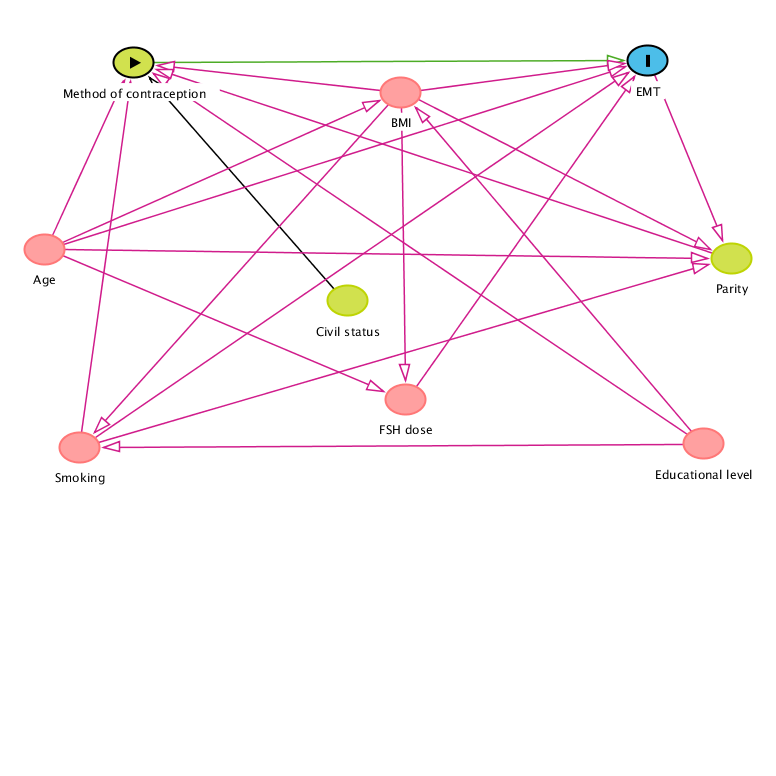


**Figure S1.** Directed acyclic graph showing potential confounding factors of the association between the use of contraceptives and endometrial thickness (EMT). Potential confounding factors included age^1^; BMI^2-5^; smoking^6-8^; educational level^9-11^ and total dose of follicle stimulating hormone. (Created with daggity.net, version 3.0)

1. Amir W, Micha B, Ariel H, Liat LG, Jehoshua D, Adrian S. Predicting factors for endometrial thickness during treatment with assisted reproductive technology. Fertil Steril. 2007 Apr;87(4):799-804.

2. Piirtola M, Jelenkovic A, Latvala A, Sund R, Honda C, Inui F, et al. Association of current and former smoking with body mass index: A study of smoking discordant twin pairs from 21 twin cohorts. PLoS One. 2018;13(7):e0200140.

3. Mosher WD, Lantos H, Burke AE. Obesity and contraceptive use among women 20-44years of age in the United States: results from the 2011-15 National Survey of Family Growth (NSFG). Contraception. 2018 May;97(5):392-8.

4. Iversen DS, Kesmodel US, Ovesen PG. Associations between parity and maternal BMI in a population-based cohort study. Acta Obstet Gynecol Scand. 2018 Jun;97(6):694-700.

5. Crosby D, O'Brien Y, Glover L, Martyn F, Wingfield M. Influence of body mass index on the relationship between endometrial thickness and pregnancy outcome in single blastocyst frozen embryo transfer cycles. Hum Fertil (Camb). 2020 Apr;23(1):32-7.

6. Sequí-Canet JM, Sequí-Sabater JM, Marco-Sabater A, Corpas-Burgos F, Collar Del Castillo JI, Orta-Sibú N. Maternal factors associated with smoking during gestation and consequences in newborns: Results of an 18-year study. J Clin Transl Res. 2022 Feb 25;8(1):6-19.

7. Rygh E, Gallefoss F, Grøtvedt L. Trends in maternal use of snus and smoking tobacco in pregnancy. A register study in southern Norway. BMC Pregnancy Childbirth. 2019 Dec 16;19(1):500.

8. Heger A, Sator M, Walch K, Pietrowski D. Smoking Decreases Endometrial Thickness in IVF/ICSI Patients. Geburtshilfe Frauenheilkd. 2018 Jan;78(1):78-82.

9. Ruokolainen O, Härkänen T, Lahti J, Haukkala A, Heliövaara M, Rahkonen O. Association between educational level and smoking cessation in an 11-year follow-up study of a national health survey. Scand J Public Health. 2021 Dec;49(8):951-60.

10. Lethbridge DJ. Use of contraceptives by women of upper socioeconomic status. Health Care Women Int. 1990;11(3):305-18.

11. Doll H, Vessey M, Painter R. Return of fertility in nulliparous women after discontinuation of the intrauterine device: comparison with women discontinuing other methods of contraception. Bjog. 2001 Mar;108(3):304-14.

**Table S1.** List of fertility clinics participating in the study.

| ***EU TE code*** | ***Fertility clinic*** |
| --- | --- |
| DK271520 | The Fertility Unit, Aalborg University Hospital, Aalborg, Denmark |
| DK257474 | The Fertility Clinic, Copenhagen University Hospital Herlev, Herlev, Denmark |
| DK269054 | Stork IVF Clinic, Copenhagen, Denmark |
| DK257482 | The Fertility Clinic, Skive Regional Hospital, Skive, Denmark |
| DK265534 | The Fertility Clinic, Horsens Regional Hospital, Horsens, Denmark |
| DK257476 | The Fertility Clinic, Department of Gynecology, Fertility, and Obstetrics, Rigshospitalet, Copenhagen University Hospital, Copenhagen, Denmark |
| DK257556 | The Fertility Clinic, Odense University Hospital, Odense, Denmark |
| DK257500 | The Fertility Unit, North Zealand Hospital, Hillerød, Denmark |

**Table S2.** Characteristics of included and excluded cycles.

|  | **Included** | **Excluded** | **Total** | **Missings / N (%)** |
| --- | --- | --- | --- | --- |
| **N (%)** | 22,464 (22.7) | 76,707 (77.3) | 99,171 (100.0) | 0 / 99,171 (0.0) |
| **Age. median (10^th^ - 90^th^ percentiles)** | 32.67 (26.73; 39.56) | 33.97 (27.24; 40.90) | 33.62 (27.10; 40.49) | 12,339 / 99,171 (12.4) |
| **BMI. median (10^th^ - 90^th^ percentiles)** | 23.77 (19.78; 30.10) | 23.72 (19.72; 30.11) | 23.73 (19.72; 30.11) | 20,229 / 99,171 (20.4) |
| **Smoking. n (%)** |  |  |  |  |
| **No** | 20,496 (91.2) | 41,237 (90.8) | 61,733 (91.0) |  |
| **Yes** | 1968 (8.8) | 4168 (9.2) | 6136 (9.0) | 31,302 / 99,171 (31.6) |
| **Educational level. n (%)** |  |  |  |  |
| **Low** | 1930 (8.6) | 3710 (7.9) | 5640 (8.1) |  |
| **Medium** | 8387 (37.3) | 16,469 (35.1) | 24,856 (35.8) |  |
| **High** | 12,147 (54.1) | 26,776 (57.0) | 38,923 (56.1) | 29,752 / 99,171 (30.0) |

**Table S3.** Analysis assessing the association between previous method of contraception and odds of achieving an endometrial thickness ≥7 mm.

|  | ***N*** | ***OR*** | ***95% CI*** | ***ORa*** | ***95% CI_a_*** |
| --- | --- | --- | --- | --- | --- |
| **LNG-IUS** | 76 | 1.00 | *Reference* | 1.00 | *Reference* |
| **CC** | 4,899 | 2.20 | (0.80; 6.07) | 3.86 | (1.40; 10.62) |
| **OCPs** | 14,535 | 3.15 | (1.15; 8.66) | 4.98 | (1.82; 13.61) |
| **POPs** | 136 | 5.79 | (1.29; 25.93) | 5.16 | (1.18; 22.67) |

Mixed effect logistic regression was used. and personal identification number (CPR) of each woman was included as a random effect. OR = odds ratio, 95% CI = 95% confidence interval, LNG-IUS = levonorgestrel intrauterine system, CC = Combined, cumulated use of contraception, OCP = combined oral contraceptive pill, POP = progestin-only pill. a: adjusted for age (continuous), BMI (continuous), smoking, educational level, total dose of follicle stimulating hormone (continuous), fertility clinic and time of contraceptive use (categorical).

**Table S4.** Sensitivity analysis assessing whether a change in cut-off values of endometrial thickness to ≤ 5 mm and ≥ 7 mm would affect the results.

|  | ***N*** | ***OR*** | ***95% CI*** | ***ORa*** | ***95% CI_a_*** |
| --- | --- | --- | --- | --- | --- |
| **No/other** | 2,723 | 1.00 | *Reference* | 1.00 | *Reference* |
| **Ever use LNG-IUS 0-3 years** | 1,249 | 0.27 | (0.14; 0.55) | 0.27 | (0.14; 0.55) |
| **Ever use LNG-IUS >3-6 years** | 926 | 0.21 | (0.10; 0.44) | 0.21 | (0.11; 0.43) |
| **Ever use LNG-IUS >6-9 years** | 72 | 0.06 | (0.01; 0.24) | 0.05 | (0.01; 0.21) |
| **Ever use LNG-IUS >9 years** | 26 | 0.08 | (0.01; 0.97) | 0.07 | (0.01; 0.78) |

Mixed effect logistic regression was used, and the personal identification number (CPR) of each woman was included as a random effect. OR = odds ratio, 95% CI = 95% confidence interval, LNG-IUS = levonorgestrel intrauterine system. a: adjusted for age (continuous), BMI (categorical), smoking, educational level, total dose of follicle stimulating hormone (continuous), and fertility clinic.

**Table S5.** Secondary analyses assessing the association between previous method of contraception and odds of achieving a clinical pregnancy.

|  |  | ***Not including no/other contraception*** | | | | ***Including no/other contraception*** | | | |
| --- | --- | --- | --- | --- | --- | --- | --- | --- | --- |
|  | ***Clinical pregnancy, n (N)*** | ***OR*** | ***95% CI*** | ***OR*_a_** | ***95% CI*_a_** | ***OR*** | ***95% CI*** | ***OR*_b_** | ***95% CI*_b_** |
| **LNG-IUS** | 13 (44) | 1.00 | *Reference* | 1.00 | *Reference* | 1.00 | *Reference* | 1.00 | *Reference* |
| **CC** | 1,049 (3,067) | 2.28 | (0.67; 7.75) | 1.16 | (0.56; 2.41) | 2.27 | (0.68; 7.63) | 1.22 | (0.59; 2.54) |
| **OCPs** | 3,023 (8,999) | 3.36 | (1.00; 11.34) | 1.13 | (0.54; 2.34) | 3.33 | (1.00; 11.13) | 1.16 | (0.56; 2.41) |
| **POPs** | 35 (76) | 6.57 | (0.99; 43.70) | 2.36 | (0.96; 5.76) | 6.48 | (0.99; 42.45) | 2.27 | (0.93; 5.54) |
| **No/other** | 531 (1,766) | - | - | - | - | 5.43 | (1.57; 18.74) | 1.07 | (0.51; 2.24) |

Mixed effect logistic regression was used, and the personal identification number (CPR) of each woman was included as a random effect. OR = odds ratio, 95% CI = 95% confidence interval, LNG-IUS = levonorgestrel intrauterine system, CC = Combined, cumulated use of contraception, OCP = combined oral contraceptive pill, POP = progestin-only pill. a: adjusted for age (categorical), BMI (categorical), smoking, educational level, total dose of follicle stimulating hormone (continuous), fertility clinic and time of contraceptive use (categorical). b: adjusted for age (categorical), BMI (categorical), smoking, educational level, total dose of follicle stimulating hormone (continuous), and fertility clinic.

**Table S6.** Secondary analyses assessing the association between ever use of LNG-IUS and odds of achieving a clinical pregnancy.

|  | ***Clinical pregnancy, n (N)*** | ***OR*** | ***95% CI*** | ***OR_a_*** | ***95% CI_a_*** |
| --- | --- | --- | --- | --- | --- |
| **No/other** | 531 (1,766) | 1.00 | *Reference* | 1.00 | *Reference* |
| **Ever use LNG-IUS 0-3 years** | 296 (854) | 1.29 | (1.04; 1.60) | 1.28 | (1.00; 1.63) |
| **Ever use LNG-IUS >3-6 years** | 216 (642) | 1.22 | (0.96; 1.54) | 1.19 | (0.93; 1.51) |
| **Ever use LNG-IUS >6-9 years** | 18 (52) | 1.14 | (0.56; 2.32) | 1.13 | (0.58; 2.20) |
| **Ever use LNG-IUS >9 years** | 8 (18) | 2.01 | (0.64; 6.37) | 2.12 | (0.74; 6.08) |

Mixed effect logistic regression was used, and the personal identification number (CPR) of each woman was included as a random effect. OR = odds ratio, 95% CI = 95% confidence interval, LNG-IUS = levonorgestrel intrauterine system. a: adjusted for age (categorical), BMI (categorical), smoking, educational level, total dose of follicle stimulating hormone (continuous), and fertility clinic.

**Table S7**. Post-hoc analyses assessing the association between previous method of contraception and odds of achieving an endometrial thickness ≥7 mm using only cycle 1 data.

|  |  | ***Not including no/other contraception*** | | | | ***Including no/other contraception*** | | | |
| --- | --- | --- | --- | --- | --- | --- | --- | --- | --- |
|  | ***N*** | ***OR*** | ***95% CI*** | ***OR*_a_** | ***95% CI*_a_** | ***OR*** | ***95% CI*** | ***OR*_b_** | ***95% CI*_b_** |
| **LNG-IUS** | 50 | 1.00 | *Reference* | 1.00 | *Reference* | 1.00 | *Reference* | 1.00 | *Reference* |
| **CC** | 2,932 | 1.12 | (0.44; 2.85) | 1.73 | (0.67; 4.47) | 1.12 | (0.44; 2.85) | 1.15 | (0.45; 2.94) |
| **OCPs** | 8,116 | 1.45 | (0.58; 3.68) | 2.04 | (0.80; 5.22) | 1.45 | (0.58; 3.68) | 1.50 | (0.59; 3.82) |
| **POPs** | 83 | 4.50 | (0.84; 24.14) | 4.08 | (0.75; 22.08) | 4.50 | (0.84; 24.14) | 4.65 | (0.86; 25.09) |
| **No/other** | 1,605 | - | - | - | - | 2.24 | (0.86; 5.79) | 2.44 | (0.94; 6.37) |

Mixed effect logistic regression was used, and the personal identification number (CPR) of each woman was included as a random effect. OR = odds ratio, 95% CI = 95% confidence interval, LNG-IUS = levonorgestrel intrauterine system, CC = Combined, cumulated use of contraception, OCP = combined oral contraceptive pill, POP = progestin-only pill. a: adjusted for age (continuous), BMI (continuous), smoking, educational level, total dose of follicle stimulating hormone (continuous), fertility clinic, and time of contraceptive use (categorical). b: adjusted for age (continuous), BMI (continuous), smoking, educational level, total dose of follicle stimulating hormone (continuous), and fertility clinic.

**Table S8.** Post-hoc analyses assessing the association between ever use of LNG-IUS and odds of achieving an endometrial thickness ≥7 mm using only cycle 1 data.

|  | ***N*** | ***OR*** | ***95% CI*** | ***OR_a_*** | ***95% CI_a_*** |
| --- | --- | --- | --- | --- | --- |
| **No/other** | 1,605 | 1.00 | *Reference* | 1.00 | *Reference* |
| **Ever use LNG-IUS 0-3 years** | 787 | 0.49 | (0.35; 0.69) | 0.47 | (0.33; 0.67) |
| **Ever use LNG-IUS >3-6 years** | 626 | 0.33 | (0.24; 0.46) | 0.31 | (0.22; 0.44) |
| **Ever use LNG-IUS >6-9 years** | 57 | 0.21 | (0.10; 0.42) | 0.19 | (0.09; 0.39) |
| **Ever use LNG-IUS >9 years** | 19 | 0.19 | (0.06; 0.58) | 0.16 | (0.05; 0.52) |

Logistic regression was used. OR = odds ratio, 95% CI = 95% confidence interval, LNG-IUS = levonorgestrel intrauterine system a: adjusted for age (continuous), BMI (categorical), smoking, educational level, total dose of follicle stimulating hormone (continuous) and fertility clinic.
